# Electrochemical Simulation of Phase I Hepatic Metabolism of Voriconazole Using a Screen-Printed Iron(II) Phthalocyanine Electrode

**DOI:** 10.3390/pharmaceutics15112586

**Published:** 2023-11-04

**Authors:** Michał Wroński, Jakub Trawiński, Robert Skibiński

**Affiliations:** Department of Medicinal Chemistry, Faculty of Pharmacy, Medical University of Lublin, Jaczewskiego 4, 20-090 Lublin, Poland; 54270@student.umlub.pl (M.W.); jakub.trawinski@umlub.pl (J.T.)

**Keywords:** metabolites, antifungal drugs, azole derivatives, screen-printed electrodes (SPE), phthalocyanine electrodes, mass spectrometry, UHPLC, HLM, principal component analysis (PCA)

## Abstract

Understanding the metabolism of pharmaceutical compounds is a fundamental prerequisite for ensuring their safety and efficacy in clinical use. However, conventional methods for monitoring drug metabolism often come with the drawbacks of being time-consuming and costly. In an ongoing quest for innovative approaches, the application of electrochemistry in metabolism studies has gained prominence as a promising approach for the synthesis and analysis of drug transformation products. In this study, we investigated the hepatic metabolism of voriconazole, an antifungal medication, by utilizing human liver microsomes (HLM) assay coupled with LC-MS. Based on the obtained results, the electrochemical parameters were optimized to simulate the biotransformation reactions. Among the various electrodes tested, the chemometric analysis revealed that the iron(II) phthalocyanine electrode was the most effective in catalyzing the formation of all hepatic voriconazole metabolites. These findings exemplify the potential of phthalocyanine electrodes as an efficient and cost-effective tool for simulating the intricate metabolic processes involved in drug biotransformation, offering new possibilities in the field of pharmaceutical research. Additionally, in silico analysis showed that two detected metabolites may exhibit significantly higher acute toxicity and mutagenic potential than the parent compound.

## 1. Introduction

Voriconazole, an antifungal medication belonging to the triazole derivatives, has been available in the European Union since 2002. It is widely utilized as a broad-spectrum antifungal, offering an enhanced therapeutic choice for addressing severe fungal infections. Voriconazole has demonstrated efficacy as a first-line treatment for invasive fungal infections such as aspergillosis or invasive fluconazole-resistant candidemia [1].

The mechanism of action of voriconazole involves potent inhibition of cytochrome P450 (CYP)-dependent 14α-sterol demethylase. By inhibiting this enzyme, voriconazole disrupts ergosterol biosynthesis and impairs the integrity and function of the fungal cell membrane. A strong CYP inhibition also increases the potential for various drug–drug interactions. Voriconazole undergoes extensive metabolic transformations, and CYP2C19, CYP2C9, and CYP3A4 are included in its metabolism. Approximately 98% of the administered dose is metabolized, and only 2% is excreted unchanged in urine [1]. The main metabolite observed, that accounts for 72% of all circulating metabolites in patients’ plasma, is an N-oxide. Other important metabolites are 4-hydroxyvoricaonazole, as well as hydroxy- and dihydroxyvoriconazole, which are products of pyrimidine hydroxylation [1]. Furthermore, as part of the clearance process these hydroxylated metabolites can undergo O-glucuronidation through hydroxyl groups, and voriconazole itself can also undergo direct glucuronidation [2,3]. Research conducted by Bourcier et al. has shown that voriconazole can serve as a substrate for UDP (uridine diphosphate glucose)-glucuronosyltransferase (UGT). After incubation with recombinant UGT enzymes, voriconazole underwent direct N-glucuronidation within the triazole ring. UGT1A4 was identified as the primary enzyme involved in this reaction.

Understanding the metabolism of drugs is crucial in pharmacological research as it impacts their effectiveness, toxicity, and pharmacokinetics. For instance, voriconazole N-oxide does not contribute to its antifungal properties [1] but has been associated with some adverse reactions such as photosensitivity and photocarcinogenicity [4]. The study of drug metabolism plays a pivotal role in optimizing therapy and minimizing adverse effects. Metabolism refers to the chemical transformations that a drug undergoes within the human body, involving various enzymatic reactions that convert the parent drug into metabolites, which may possess different pharmacological properties. Biotransformation can significantly influence drug efficacy, bioavailability, and the overall safety profile. Studying drug metabolism aids in elucidating the potential drug–drug interactions and identifying factors that may influence individual variation in drug response, thus guiding clinical decision-making, such as dosage adjustments in patients with impaired liver function or interactions with co-administered medications [5,6]. The study of biotransformation also provides a valuable tool for identifying active metabolites, which can be further explored to enhance drug therapy and advance research in the field [7].

The liver is the most important organ for drug metabolism, but biotransformation in other tissues, such as the kidneys, intestines, lungs, brain, nasal epithelium, and skin, can also occur [5,8]. Liver abundance in cytochrome P450 enzymes plays a crucial role in catalyzing the majority of the metabolic reactions in the organisms. To study these metabolic processes in a controlled laboratory setting, researchers often utilize a simplified in vitro model system, such as Human Liver Microsomes (HLMs), that mimics the metabolic processes occurring in the liver. HLMs are derived from the liver’s endoplasmic reticulum through differential high-speed centrifugation. This technique allows for the isolation of the subcellular fractions that contain important enzymes involved in hepatic drug metabolism, including cytochrome P450s and UGTs (UDP-glucuronosyltransferases) [9].

Despite its unequivocal usefulness in drug metabolism studies, some disadvantages associated with the use of HLM also exist. Due to the absence of enzymes like N-acetyltransferases (NAT), glutathione-S-transferases (GST), and sulfotransferases (SULT) and necessary cofactors, the formation of some metabolites that are present in vivo may not be achieved using this approach. The noticeable reduction in reaction efficiency concerning the incubation duration additionally curtails the utility of this approach as a simple and effortless source of metabolites [8]. Additionally, the presence of biological interferences, such as the cellular matrix, phospholipids, and proteins, requires a complex process and a time-consuming workflow to isolate metabolites. Some highly reactive phase I metabolites can rapidly degrade or form irreversible bonds with cellular macromolecules, including proteins and even DNA before phase II conjugation occurs, making their detection in complex biological matrices difficult. Thus, alternative methods are being sought to study metabolism.

Electrochemistry presents a novel perspective in metabolism research, focusing on redox reactions, where the exchange of electrons between molecules plays a crucial role. These reactions account for most of the reactions in the body, including dehydrogenations, hydrolyses, reductions, or oxidations, particularly during the first phase of biotransformation where CYP450 enzymes play a significant role [10]. The utilization of electrochemistry (EC) in metabolism studies offers a promising approach for the synthesis and analysis of oxidative drug metabolites. The versatility of EC reactions allows for a wide range of oxidation pathways, which can mimic the activities of CYP450 enzymes in living organisms [11,12]. However in EC synthesis, depending on the experimental conditions, the nature of the electrode, the presence of mediators or electrochemically generated reactive species, and other factors, the electrochemical oxidation of a drug compound can follow distinct reaction pathways [12].

Screen-printed electrodes (SPE) are planar devices featuring a three-electrode configuration in miniaturized format. They consist of plastic substrates coated with precisely controlled layers of electroconductive and insulating inks. The introduction of this technology has facilitated the mass production of inexpensive, disposable electrodes, enabling effective execution of electrochemical experiments. SPEs have a wide range of applications. They play a crucial role in environmental monitoring, helping to detect and quantify pollutants [13], in microbiology [14] and in food industry analysis, and are also essential in clinical diagnostics, enabling the measurement of biomarkers and disease-related molecules in blood and urine samples [15]. Although, only carbon SPEs have been successfully employed to mimic the metabolism of eugenol [16], raloxifene [17], and paracetamol [18], efficiently generating both phase I and phase II metabolites of tested drugs. Carbon electrodes remain among the most commonly employed choices. Nevertheless, carbon electrodes modified with phthalocyanines are gaining increasing attention. Thanks to their conductivity and catalytic properties, numerous novel sensors have been developed using various phthalocyanines as modifiers for the analysis of diverse substances. Among the most commonly employed transition metals in phthalocyanine electrodes were Co(II), Fe(II), Fe(III) Cu(II), Ni(II), and Mn(II). These materials have garnered preference in analytical applications due to their exceptional catalytic capabilities. One of their great advantages is their ability to operate efficiently over a broad range of pH values and exhibit wide working potentials in both anodic and cathodic directions. The broad electrochemical window not only significantly enhances peak intensity, but also lowers the peak potential, signifying improved kinetic parameters. Consequently, analytes can be detected at lower potentials in voltammetric studies. However, to the best of our knowledge, the application of phthalocyanine SPEs in metabolic studies has not been investigated.

In this investigation, we explored the hepatic metabolism of voriconazole established by utilizing HLM assay in combination with LC-MS. Subsequently, based on the obtained results, we optimized electrochemical parameters to simulate biotransformation reactions using various SPE electrode materials, including innovative ones. The utilization of multivariate chemometric analyses, such as Principal Component Analysis (PCA), enabled us to select the optimal experimental conditions for the electrochemical experiments. Preliminary analysis of the metabolites, acute toxicity to rodents, mutagenicity, and developmental toxicity were carried out using an in silico approach.

## 2. Materials and Methods

### 2.1. Chemicals and Reagents

Voriconazole was obtained in a commercially available pharmaceutical formulation—Voriconazol Polpharma 200 mg tablets (Polpharma, Gdańsk, Poland). Five tablets were grounded in a mortar, and then weighed. The equivalent of 87.25 mg of voriconazole was ultrasonicated for 5 min in a 25 mL volumetric flask with 15 mL of acetonitrile, and then filled up to the mark with the same solvent and centrifuged. The supernatant was collected and used as a stock solution (10 mM) for biological and electrochemical experiments.

Acetonitrile for LC-MS, water for LC-MS and water for LC were purchased from Witko (Łódź, Poland) and 99% formic acid (MS grade) was obtained from Avantor Performance Materials Poland S.A. (Gliwice, Poland). Hydrochloric acid and sodium hydroxide were purchased from POCh (Gliwice, Poland). Sodium phosphate dibasic anhydrous salt was purchased from Sigma-Aldrich (St. Louis, MO, USA).

The toxic potential of voriconazole and its identified hepatic metabolites was evaluated using an in silico approach using ACD/Labs Percepta 14.0.0 (2015 Release, Advanced Chemistry Development, Inc., Toronto, ON, Canada), the Toxicity Estimation Software Tool (T.E.S.T., Version 5.1.1, EPA, Washington, DC, USA), and Vega platform (Version: 1.1.5-b48, calculation core version: 1.2.8, Istituto di Ricerche Farmacologiche Mario Negri, Milan, Italy). Exploration of the obtained results using the PCA was performed with the use of R 4.1.0 software (GNU Project).

### 2.2. In Vitro Metabolism Simulation by HLM

For the biotransformation experiment, a stock solution of voriconazole in acetonitrile was diluted with ultrapure water to working concentrations (1 mM). Human liver microsomes (HLMs) fraction was used for conducting the in vitro biotransformation study (typical isolation protocol of this subcellular fraction has been described by Hoensch et al.) [19]. The incubation mixture contained 0.05 mM of substrate, 50 mM of phosphate buffer (pH 7.4), 20 mM of NADPH, and 0.5 mg mL^−1^ HLM. After a 2 min pre-incubation period at 37 °C under gentle, constant shaking in an Eppendorf ThermoMixer C equipped with Eppendorf ThermoTop (Eppendorf AG, Hamburg, Germany), the metabolic reactions were initiated by the addition of NADPH. Following an incubation time of 0, 60, 120, and 180 min, the reaction was stopped by adding 40 uL of ice-cold acetonitrile–methanol mixture (1:1). After precipitation, the samples were subjected to centrifugation at 16,000 rpm for 10 min at 4 °C. Subsequently, 40 μL of the supernatants were transferred into the vials for LC-MS analysis. The procedure was replicated for the negative control samples, with the only difference being the exclusion of the NADPH solution.

### 2.3. Electrochemical Studies

An electrochemical measuring instrument, the modular potentiostat/galvanostat Autolab/PGSTAT302N (Metrohm Autolab, Utrecht, Netherlands), controlled by the Nova 2.1.5. software was used for constant potential amperometry experiments. The electrochemical (EC) behavior of voriconazole was investigated using various working electrodes, namely platinum (Pt), gold (Au), glassy carbon (GC), Iron(II) phthalocyanine (FePH), and Copper(II) phthalocyanine (CuPH) SPE working electrodes. The specific electrode models used were 550BT, 220BT, 110, 110FePH, and 110CuPH, respectively (Metrohm DropSens). In Pt and Au SPEs, platinum or gold is used as both the working electrode and the auxiliary electrode, respectively. Silver serves as the reference electrode. In the case of glassy carbon (GC), Iron(II) phthalocyanine (FePH), and Copper(II) phthalocyanine (CuPH) SPEs, carbon is used as the auxiliary electrode, while silver serves as the reference electrode. The electrochemical behavior of voriconazole has been examined by conducting experiments at potentials of 0.8, 1.0, 1.2, 1.4, 1.6, and 1.8 V for 15 min, and interval time 0.5 s. The supporting electrolyte was composed of a phosphate buffer with a pH of 7.4, mixed with acetonitrile in a 99:1 (*v*/*v*) ratio. The working solution, which contained voriconazole at a concentration of 0.025 mM, was created by diluting a 10 mM stock solution in this electrolyte. The conditions for performing chromatography were optimized based on previous studies. All the experiments were conducted at room temperature, and an 80 µL drop of the solution was placed on the surface of the SPE to cover all tested electrodes.

### 2.4. LC-MS Analysis

The LC–MS/MS analysis was performed using an Agilent high-resolution Q-TOF system series 6520 with electrospray ionization source (ESI) and a UHPLC system series 1290. A Kinetex-C18 (2.1 × 50 mm, dp = 1.8 μm) reversed-phase chromatographic column (Phenomenex, Torrance, CA, USA) was employed for analysis. The MassHunter workstation software version B.06.00 (Agilent Technologies, Santa Clara, CA, USA) was employed for control of the LC-MS system, data acquisition, qualitative, and quantitative analysis. The optimization of instrument conditions started from the tuning of the MS detector in a positive mode in an extended dynamic range (2 GHz). To ensure accuracy in mass measurements, a reference mass correction was implemented by using lock masses of 121.050873 and 922.009798. Detailed information regarding the chromatographic and spectrometric parameters can be found in Supplementary Materials Table S1.

### 2.5. Data Preprocessing and Chemometric Analysis

Each experiment was conducted with five replicates, including samples taken at the optimal incubation time with HLM (after 120 min of incubation), control samples (HLM without NADPH), and samples of EC experiments performed on each investigated electrode material at their respective optimal potentials (FePH: 1.0 V, CuPH: 1.4 V, Au: 1.6 V, Pt: 1.2 V, and GC: 1.4 V). This resulted in a total of thirty-five samples across seven different experiments for the tested drug. High-resolution LC-MS analysis was executed in TOF (MS) mode for all the samples, documenting their individual chromatographic and spectral profiles. Elimination of background ion noise in the data and the identification of characteristic ions associated with the degradation products of voriconazole were accomplished by employing the molecular feature extraction (MFE) algorithm provided by Mass Hunter Qualitative Analysis software version B.06.00 (Agilent Technologies, Santa Clara, CA, USA). To optimize the MFE parameters for accurate feature extraction, the following settings were applied: the minimum abundance was set at 5000 counts for the compound filter, a minimum of one ion was required for the identification of compound, and the used isotope model was: common organic molecules with peak spacing tolerance 0.0025 *m*/*z*. The multivariate chemometric analyses were performed with Mass Profiler Professional (MPP) software version 12.61 (Agilent and Strand Life Sciences Pvt. Ltd., Santa Clara, CA, USA).

## 3. Results and Discussion

### 3.1. Optimization of LC-MS Method and Electrochemical Experiments

The chromatographic parameters were optimized to reduce the time that the analysis took and to achieve effective separation of the generated products. Ultimately, the selected eluting solvents were aqueous formic acid (0.1%) and acetonitrile. Separation was accomplished using a gradient profile described in the supplementary material Table S1.

The preliminary study of voriconazole behavior on SPEs was conducted using various pH levels of electrolytes and acetonitrile content in a water medium. Subsequently, to perform the EC experiments, a solution comprising a 99:1 (*v*/*v*) mixture of phosphate buffer at pH 7.4 and acetonitrile was employed.

### 3.2. Biotransformation of Voriconazole

**Figure 1:**
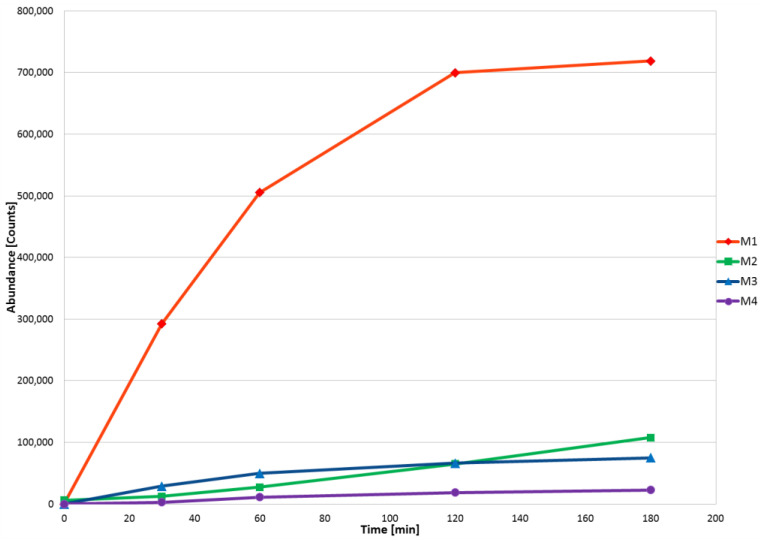
Formation of voriconazole metabolites in HLM incubation.

The kinetics of voriconazole in in vitro biotransformation was assessed using a HLM assay, focusing on the abundance of the parent ion. The study encompassed a range of incubation times (0–180 min) and demonstrated moderate metabolism of the analyzed drug. After 60 min of incubation with HLM, the formation rates of M3 and M4 significantly slowed down. In the case of M1, its formation decelerated after 120 min of incubation. In contrast, M2 increased significantly proportionally throughout the entire incubation period. Taking this into account, to perform all chemometric and qualitative analyses, a time of 120 min for HLM incubation with voriconazole was selected. Evolution profiles of voriconazole metabolites obtained during incubation with HLM are presented in Figure 1.

### 3.3. Multivariate Comparison of HLM Metabolites and Electrochemical Products

Application of the EC techniques in the simulation of metabolic reactions has gained considerable popularity in the past decade. Nevertheless, the establishment of universal, optimal conditions that would enable mimicking the biotransformation redox reactions is not possible. The reasons for the current state of things are mainly the different chemical interactions between the drugs’ molecules and various working electrode materials. Based on the EC simulation of the phase I metabolism of 21 drugs, Pedersen et al. concluded that the three most popular electrodes gave very similar efficiency in the terms of the percentage of formed metabolites (GC—78%, Pt and Au—72%) [20]. However, despite the close overall performance, the qualitative metabolic profiles strongly depended on the electrode chemistry, for instance only two of the six lidocaine metabolites were formed on the Au, while on Pt five were formed (opposite result was obtained in case of 5-methoxy-N,N-diallyltryptamine).

**Figure 2:**
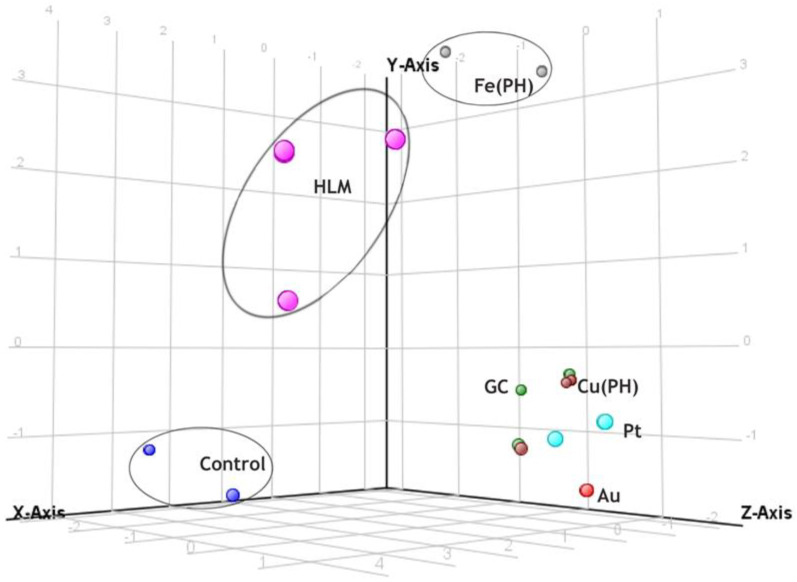
3D principal component analysis plot of electrochemical and biological HLM profiles of voriconazole.

Taking this into account, it is difficult to predict which electrode material could provide the most relevant metabolic profile. Therefore, multivariate chemometric analysis was carried out to assess qualitative and quantitative differences in the recorded profiles of voriconazole metabolites obtained through two different methods: biological and electrochemical experiments. In this study, a total of thirty-five chromatograms were collected in TOF (MS) mode and were aligned using MPP software, resulting in 108 distinct entities. Applying build-in MPP filtration techniques, including filtering by flags and sample abundance, followed by setting the threshold on a level no less than 4.0, and a one-way ANOVA test (*p* = 0.05), resulted in the selection of a final set of 12 entities for further chemometric analysis. In the presented PCA (Figure 2), 84.2% of the total variance was explained by the first three principal components (PCs). The clear separation of control samples from other experimental samples confirms the occurrence of metabolic reactions. Notably, the PCA results indicated that FePH electrodes provided the closest simulation to HLM. In contrast, all experiments conducted with other types of electrode materials were clustered together and were situated farther from the HLM samples compared to the FePH samples. Additionally, the FePH samples were positioned even farther away from the control samples than samples from other EC experiments. Among all the electrode material tested, Au and Pt performed the worst in terms of reflecting the results of the experiment with HLM.

M1 and M3 are formed with the best efficiency on the Fe(PH) electrode, while all electrodes showed similar efficiencies in forming M4 (Figure S1). M2 is formed best on the Fe(PH) electrode, with the exception of the Au electrode (Figure S2). The Fe(PH) electrode performed more favorably due to the lower quantity of non-metabolite electrochemical transformation products formed. Electrochemical reactions on most electrodes resulted in the formation of two non-metabolite products, which are the outcome of mono-oxygenation. Products with an *m*/*z* value of 366.1172 (retention time of 4.40) exhibited significant formation on Fe(PH) SPE. In contrast, another non-metabolite product with an *m*/*z* value of 366.1172 (retention time of 4.23) was formed on the Au and Pt SPEs with high efficiency. Overall, the Au and Pt electrodes did not prove to be as effective in generating metabolites M1 and M3, yet transformation products were produced in relatively large quantities on them. Extracted ion chromatograms (EICs) for optimal conditions for obtaining metabolites with *m*/*z* values of 366.1172 and 224.0630 for each type of electrode are presented in the Supplementary Materials.

### 3.4. Metabolite Identification and Transformation Pathway

In the present investigation, four products of voriconazole hepatic metabolism were found and identified through the application of high-resolution mass spectrometry. Table 1 summarizes the fragmentation pattern of voriconazole and its metabolites, while MS/MS spectra are presented in the Supplementary Materials.

Three of the identified metabolites resulted from the oxidation reactions of voriconazole. Two of them are hydroxy derivatives, in which hydroxylation took place at positions 1 or 4, respectively, of the aliphatic ring, and the third resulted from the N-oxidation at the pyrimidine ring, which constitutes the primary metabolite. The fourth product arose from the decomposition of voriconazole. M2 and M4 are metabolites registered for the first time in human hepatic metabolism.

The MS/MS spectrum of voriconazole (350.1223 *m*/*z*) is depicted in Figure S3, revealing its fragmentation pathway. This pathway begins with the elimination of the triazole moiety (281.0898 *m*/*z*). An alternative route starts with the detachment of 4-ethyl-5-fluoropyrimidine (127.0663 *m*/*z*) from the residue, which consists of an ethanol molecule with triazole and difluorophenyl substituents (224.0630 *m*/*z*). The loss of the triazolomethyl moiety from the 224.0630 *m*/*z* fragment results in the appearance of a fragment with an *m*/*z* value of 141.0144 in the MS/MS spectrum. Additionally, a fragment with an *m*/*z* value of 70.0404, representing triazole, is visible in the MS/MS spectrum. The elimination of a hydroxyl group from the buthan-2-ol chain in voriconazole and the 281.0898 *m*/*z* fragment leads to the presence of a minor peak in the spectrum with *m*/*z* values of 332.1119 and 263.0791, respectively.

M1 (366.1153 *m*/*z*) is an N-oxide, in which oxidation takes place along the pyrimidine ring. The primary peak in the MS/MS spectrum (Figure S4) appears at an *m*/*z* value of 224.0624 and is also present in parent spectrum. This indirectly suggests that both triazole and difluorophenyl moieties remain unchanged. This fragment was formed as a result of the detachment of a hydroxylated fragment of 6-ethyl-5-fluoropyrimidine which is also visible on the spectrum in the form of a prominent peak at *m*/*z* with value of 143.0610. The consecutive largest peak is at 126.0580 *m*/*z*, representing the same fragment after the loss of oxygen, indicating an impermanent oxygen connection, which is indirect evidence of structure oxidation in the form of N-oxide. Further fragmentation of this fragment leads to subsequent removal of one carbon and one fluorine atom from pyrimidine moiety. Fragments at 115.0660 *m*/*z* and 95.0604 *m*/*z* represent 5-ethyl-4-fluoro-imidazole and 5-ethyl-imidazole, respectively. Moreover, a fragment at 348.1099 *m*/*z* demonstrates the easy removal of a hydroxyl group from M1. Two peaks at 297.0804 *m*/*z* and at 279.0742 *m*/*z* represent structures after subsequent triazole and the hydroxyl group’s detachment from M1, indicating the presence of oxidation within the remaining structures.

Although the fragmentation spectra do not provide a definitive location for the formation of the N-oxide on the nitrogen atom within the pyrimidine ring, we can assume its location based on prior findings [21]. Voriconazole N-oxide is formed through two main metabolic pathways. According to the Schulz et al. studies, for voriconazole, N-oxidation are mainly contributed CYP2C19, CYP3A4, and CYP2C9, responsible for 62%, 48%, and 36% of N-oxidation, respectively [22]. In another study, the involvement of flavin-containing monooxygenase (FMO) enzymes in the formation of N-oxide was elucidated, with FMO3 emerging as the primary enzyme responsible for this metabolic process [23].

M2 with an *m*/*z* value of 224.0621 corresponds to the compound 1-(2,4-difluorophenyl)-2-(1H-1,2,4-triazol-1-yl)ethan-1-one. M2 was formed through the chain disruption, leading to the detachment of the ethyl-fluoropyrimidine moiety from voriconazole. Fragmentation of M2 starts with the detachment of the triazole moiety, visible on the spectrum (Figure S5) at an *m*/*z* value of 70.0410, from the fragment with 155.0312 *m*/*z*. Subsequent fragmentation leads to the formation of difluoromethylbenzene, which is the primary peak in the spectrum with an *m*/*z* value of 127.0354. The loss of two carbon atoms from this fragment leads to the formation of difluorocyclopentane (101.0181 *m*/*z*). Two small peaks at *m*/*z* values of 82.0396 and 83.0470 correspond to methyltriazole and an alkyl chain formed from the fragmentation of the 155.0312 *m*/*z* fragment, respectively. While CYP enzymes do not frequently catalyze the cleavage of carbon–carbon bonds, there are documented cases in which theses enzymes are responsible for such reactions [24].

The main peak in the MS/MS spectrum of M3 (366.1144 *m*/*z*) (Figure S6) is observed at 297.0848 *m*/*z*, which corresponds to the 281.0898 *m*/*z* fragment from the parent MS/MS spectrum that underwent hydroxylation. The 224.0626 *m*/*z* fragment, which is also present in the parent’s MS/MS spectrum, proves that the difluorophenyl and triazole fragments remained unchanged. The detachment of the 224.0626 *m*/*z* fragment from M3 and the loss of a hydroxyl group are represented by the last visible peak in the MS/MS spectrum (125.0509 *m*/*z*). The information derived from spectral data allows us to associate M3 with the well-documented characteristics of the related metabolite, thereby enabling us to confidently assert that our observed product aligns with a known product of hydroxylation at the 4th position of the buthan-2-ol. According to Murayama et al., the formation of 4-hydroxyvoriconazole is primarily catalyzed by CYP3A4 [25].

The second hydroxy product of voriconazole metabolism, M4, is a new compound not previously described as a human liver metabolite in the literature. In this case, hydroxylation took place at position 1 of buthan-2-ol. The fragmentation of M4 begins with the detachment of the triazole moiety. The ion at 297.0834 *m*/*z*, present in its spectrum (Figure S7), excludes hydroxylation within the triazole moiety. Subsequent dehydroxylation of this fragment results in an ion at 281.0886 *m*/*z*. This indicates that an additional oxygen atom underwent feasible elimination, suggesting that hydroxylation took place at the proposed location. A distinctive feature of the M3 spectrum is the absence of the 224.0624 *m*/*z* ion but the presence of the 240.0596 *m*/*z* ion, which indicates hydroxylation within the fragment that remained after the disconnection of ethylpyrimidine. Additionally, the ion with an *m*/*z* value of 127.0654 indicates the lack of hydroxylation within the detached fragment. A similar type of reaction connecting the formation of products M2 and M4 suggests the possibility that the same isozyme may have been involved in the creation of M4.

**Figure 3:**
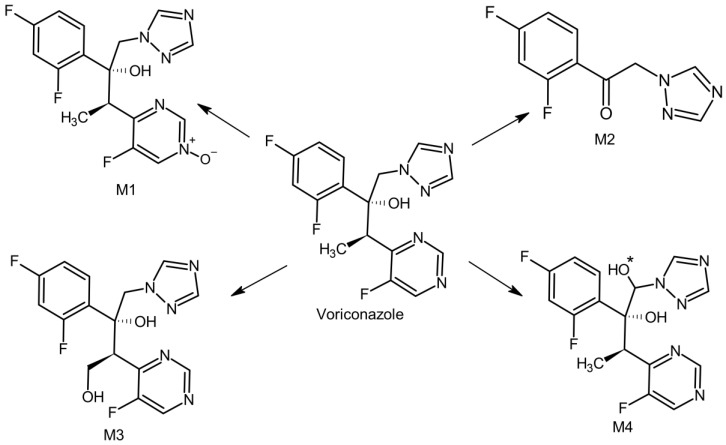
The hepatic metabolic pathway of voriconazole (*—undefined stereoisomerism).

Our findings are consistent with previous reports by Schultz et al., who also did not observe the formation of a voriconazole metabolite that undergoes hydroxylation within the pyrimidine ring in HLM assay [22]. The absence of this metabolite can be attributed to a pathway independent of CYP enzymes and FMO. Consequently, the absence of this metabolite negates the presence of the dihydroxylated product of voriconazole. The metabolites of voriconazole obtained in this study are presented in Figure 3.

**Figure 4:**
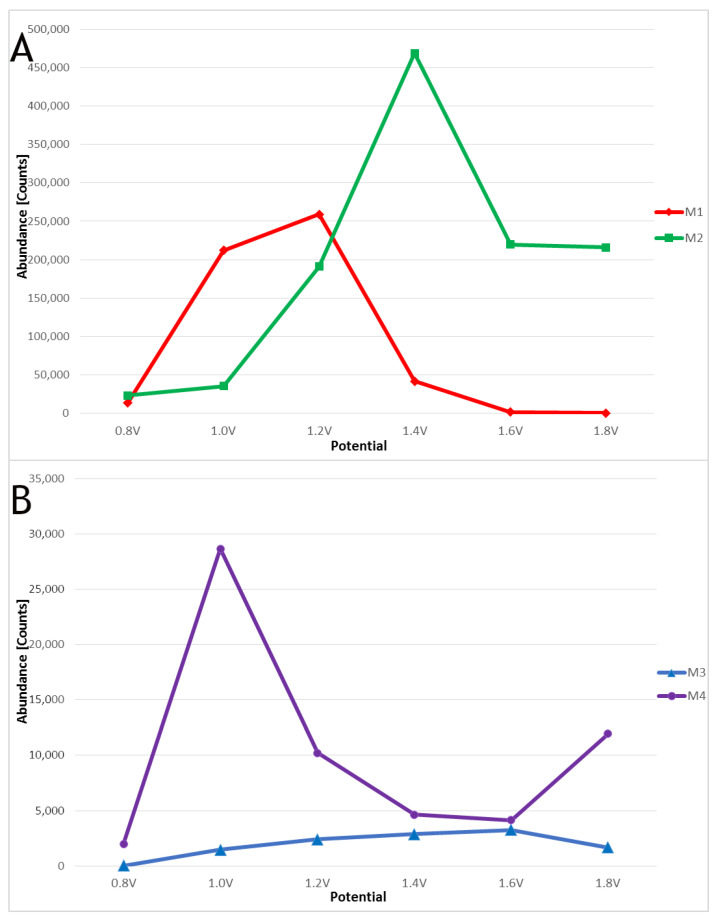
Evolution profiles of major (**A**) and minor (**B**) voriconazole metabolites in EC method.

Based on the PCA results, the FePH electrode was chosen as the best for forming voriconazole metabolites. All of the metabolites formed during the HLM assay were successfully obtained in the electrochemical experiments using the FePH SPE. Optimization experiments revealed that M2 is the predominant electrochemical product, with its formation peaking at a potential value of 1.4 V. With a further increase in potential, the efficiency of its formation begins to decrease, most likely due to increased decomposition of the resulting electrochemical product. The most readily formed hydroxylated derivative is the M1, which, in the potential range of 1.0 to 1.2 V, is the easiest electrochemical product to form, exhibiting its optimal potential at 1.2 V. M3 and M4, similarly to metabolic samples, are produced in EC experiments with notably lower efficiency. M3 has an optimal potential of 1.0 V but also shows an increased synthesis efficiency around 1.8 V, while M4 has an optimal synthesis potential of 1.6 V. Evolution profiles of metabolites formed in EC methods are presented in Figure 4.

### 3.5. Toxicity

In terms of reliability, in vivo and in vitro methods of toxicity monitoring are still considered as the most reliable. Nevertheless, taking into account their high cost, time-consuming nature, and, as is particularly important nowadays, ethical considerations, in silico methods are often used at the preliminary stage of research. Numerous studies proved the high reliability of the computational methods; however, it should be noted that their prediction quality depends both on the applied training set (and, consequently, their applicability domain) and calculation method—statistical methods can provide better results than rule-based ones [26,27,28].

Acute toxicity to rodents (LD_50_) was calculated using Percepta (six models were applied: oral, subcutaneous, intravenous, intraperitoneal for mice, and oral, intraperitoneal for rats) and T.E.S.T. (rat oral model using the nearest neighbor method with active fragment constraint was applied) software. The calculated results are shown in Table S2. In order to facilitate the interpretation of the obtained data, PCA was performed. Application of this chemometric technique enabled visualization of relationships between the analyzed compounds and the toxicity models. As shown in Figure S8A, there are three groups of correlated variables: Percepta Mouse IV—Mouse SC—Rat IP, Percepta Mouse IP—T.E.S.T. Rat OR, and Percepta Rat OR—Mouse OR. The analyzed compounds presented significantly divergent properties: M4 was the least toxic (toxicity, as expressed in mg/kg, decreases with the increasing LD_50_ values) according to the first group of models, M3 to the second group, and M1 to the third group. Although both the parent compound and M2 were generally more harmful than the aforementioned compounds, the toxic properties of M2 were definitely the highest according to all models.

Mutagenicity was calculated using five models: one provided by Percepta and T.E.S.T. (Consensus model with active fragment constraint), and three by the Vega Platform (Consensus version 1.0.3, ISS version 1.0.2 and KNN/Read-Across version 1.0.0). The obtained data are shown in the supplementary material Table S3. Similarly to the rodents toxicity, PCA was also performed in this case. As shown in Figure S8B, there are two relatively highly correlated groups of variables: Vega KNN/Read-Across and T.E.S.T. Consensus—Vega ISS—Percepta. In general, the metabolites M3 and M4 can be viewed as more mutagenic than voriconazole (also M2, however to a lesser extent) according to the first group of models, and M1 according to all the applied models (particularly to the second group of models). At this point it is worth noting that observed photomutagenic and photocarcinogenic effects are attributed, by some authors, to voriconazole N-oxide. On the other hand, other studies did not confirm those findings, suggesting that neither the parent compound, nor its major metabolite, is responsible for such adverse effects. Such findings may indicate that those toxic properties come from action of other biotransformation products, which corresponds with our results.

Developmental toxicity was estimated using two models: Consensus (with active fragment constraint) provided by T.E.S.T. and CAESAR (version 2.1.7) provided by the Vega platform. The majority of the studied compounds probably possess toxic properties (Table S4). Only M2 was defined as non-toxic by both applied models. These results are generally consistent with the literature; although studies on reproductive and developmental toxicity of voriconazole were not conducted in humans, its application during pregnancy must be avoided unless the benefit to the mother clearly outweighs the potential risk to the fetus (toxicity was confirmed in the animal studies) [29].

## 4. Conclusions

In this study, human hepatic metabolism of voriconazole was investigated and identification of its four metabolites was conducted, including two new metabolites, specifically (1-(2,4-difluorophenyl)-2-(1H-1,2,4-triazol-1-yl)ethan-1-one and 1-hydroxyvoriconazole). EC experiments conducted on SPEs were optimized to mimic biotransformation reactions. The results showed that the FePH SPE was the most effective in mimicking these reactions, allowing us to obtain all metabolites formed during incubation with HLM. This underscores the remarkable utility of SPEs in metabolism studies, as electrode processes on these electrodes enable us to successfully replicate the oxidation reactions that occur during incubation with HLMs. Notably, this approach offers significant advantages, including its speed and relatively low cost, positioning electrode processes on SPEs as valuable supplements to the commonly employed procedures for studying metabolic reactions, enabling the acquisition of specific metabolites. Furthermore, this study highlights the promising potential of phthalocyanine SPE in the realm of metabolic research, presenting them as a forward-looking choice in this field.

In silico toxicity analysis showed that most of the identified metabolites are probably less toxic to rodents than the parent compound. Only M2 was defined as significantly more harmful, which may indicate the need for further research on its toxic properties. On the other hand, the majority of the metabolites possess higher mutagenic potential than voriconazole. In this case M1, whose significant mutagenicity comes from the N-oxide group, deserves special attention.

## Figures and Tables

**Table 1:** Accurate mass, elemental composition, and proposed structures of the analyzed compounds.

Name	Retention Time [min]	Mass [*m*/*z*]		Mass Error [ppm]	Molecular Formula [M+H]^+^	Fragmentation MS/MS	
		Measured	Theoretical			Mass [*m*/*z*]	Ion Formula [M+H]^+^
Voriconazole	5.00	350.1223	350.1223	0	C_16_H_15_F_3_N_5_O	332.1119 281.0898 263.0791 224.0630 141.0144 127.0663 70.0404	C_16_H_12_F_3_N_5_ C_14_H_12_F_3_N_2_O C_14_H_10_F_3_N_2_ C_10_H_8_F_2_N_3_O C_7_H_3_F_2_O C_6_H_8_FN_2_ C_2_H_4_N_3_
M1	3.88	366.1164	366.1172	2.19	C_16_H_15_F_3_N_5_O_2_	348.1099 297.0804 279.0742 251.0797 224.0620 155.0307 143.0610 126.0580 115.0660 95.0604 82.0397 70.0403	C_16_H_13_F_3_N_5_O C_14_H_12_F_3_N_2_O_2_ C_14_H_10_F_3_N_2_O C_13_H_10_F_3_N_2_ C_10_H_8_F_2_N_3_O C_8_H_5_F_2_O C_6_H_8_FN_2_O C_6_H_7_FN_2_ C_5_H_8_FN_2_ C_5_H_7_N_2_ C_3_H_4_N_3_ C_2_H_4_N_3_
M2	3.15	224.0621	224.0630	4.02	C_10_H_8_F_2_N_3_O	155.0312 127.0354 101.0181 83.0470 82.0396 70.0410	C_8_H_5_F_2_O C_7_H_5_F_2_ C_5_H_3_F_2_ C_5_H_7_O C_3_H_4_N_3_ C_2_H_4_N_3_
M3	3.25	366.1144	366.1172	7.65	C_16_H_15_F_3_N_5_O_2_	297.0848 224.0626 125.0509	C_14_H_12_F_3_N_2_O_2_ C_10_H_8_F_2_N_3_O C_6_H_6_FN_2_
M4	4.03	366.1161	366.1172	3.00	C_16_H_15_F_3_N_5_O_2_	297.0834 281.0886 240.0596 127.0654	C_14_H_12_F_3_N_2_O_2_ C_14_H_12_F_3_N_2_O C_10_H_8_F_2_N_3_O_2_ C_6_H_8_FN_2_

## Data Availability

Data are contained within the article or Supplementary Materials.

## Supplementary Materials

The following supporting information can be downloaded at: https://www.mdpi.com/article/10.3390/pharmaceutics15112586/s1, Table S1: LC-MS parameters; Table S2: Acute toxicity of voriconazole and its metabolites to rodents (LD_50_ [mg/kg]); Table S3: Mutagenicity (probability of positive Ames test) of voriconazole and its metabolites (red—positive, green—negative); Table S4: Developmental toxicity of voriconazole and its metabolites (red—toxic, green—non-toxic; Figure S1. Overlaid EIC chromatograms of m/z 366.1172 obtained from the examined electrodes; Figure S2. Overlaid EIC chromatograms of m/z 244.0630 obtained from the examined electrodes; Figure S3: MS/MS spectrum and fragmentation pattern of voriconazole; Figure S4: MS/MS spectrum and fragmentation pattern of M1; Figure S5: MS/MS spectrum and fragmentation pattern of M2; Figure S6: MS/MS spectrum and fragmentation pattern of M3; Figure S7: MS/MS spectrum and fragmentation pattern of M4; Figure S8. Comparison of toxicity of metabolites of voriconazole by PCA; IP—Intraperitoneal, IV—Intravenous, OR—Oral, SC—Subcutaneous.
