# Supplementary material for: Electrochemical Simulation of Phase I Hepatic Metabolism of Voriconazole Using a Screen-Printed Iron(II) Phthalocyanine Electrode
Source: Pharmaceutics. 2023 Nov 4;15(11):2586. doi: 10.3390/pharmaceutics15112586 (PMC10674253; doi:10.3390/pharmaceutics15112586)
Supplement: Supplementary file 1 [file pharmaceutics-15-02586-s001.zip › pharmaceutics-2665069-supplementary.pdf]

Supplementary Materials: Electrochemical Simulation of Phase I Hepatic Metabolism of Voriconazole Using a Screen-Printed Iron(II) Phthalocyanine Electrode

Michał Wroński, Jakub Trawiński and Robert Skibiński *

Table S1. LC-MS parameters.

Device	Parameter	Value
LC	Solvents	A – acetonitrile<br>B – 0.1% aqueous solution of HCOOH
	Gradient	5% A to 80% A
	Analysis time	9 min
	Post-time equilibration	2.50 min
	Flow rate	0.3 mL min^{-1}
	Injection volume	2 μL
	Column temperature	35 °C
MS	Ion source	Electrospray (ESI)
	Mode	Positive
	Source temperature	300 °C
	Drying gas flow	10 L min^{-1}
	Nebulizer pressure	40 psig
	Capillary voltage	3500 V
	Fragmentor voltage	140 V
	Skimmer voltage	65 V
	Octopole voltage	750 V
	Mass range	90–950 m/z
	Acquisition rate	1.5 spectra s^{-1}

Table S2. Acute toxicity of voriconazole and its metabolites to rodents (LD_{50} [mg/kg]).

Compound	T.E.S.T. Rat OR	Mouse IP	Mouse OR	Mouse IV	Mouse SC	Rat IP	Rat OR
Vor	779,1	1000	650	330	5000	490	740
VM1	796,85	1400	1400	280	5600	520	800
VM2	330,64	740	910	220	2200	410	760
VM3	814,78	1200	730	390	6000	600	820
VM4	732,9	1300	1000	450	13000	720	760

OR – oral; IP – intraperitoneal; IV – intravenous; SC – subcutaneous

Table S3. Mutagenicity (probability of positive Ames test) of voriconazole and its metabolites (red – positive, green – negative).

Compound	T.E.S.T. Consensus[a]	Percepta[a]	Vega Consensus[b]	Vega ISS[c]	Vega KNN/Read-Across[c]
Vor	0,76	0,13	-0,3	0	0
VM1	0,87	0,28	0,1	1	1
VM2	0,84	0,22	-0,1	0	1
VM3	0,75	0,16	0	0	1
VM4	0,82	0,09	0	0	1

a – mutagenic > 0,5; b – mutagenic >= 0; c – 0 = non-mutagenic, 1 = mutagenic

Table S4. Developmental toxicity of voriconazole and its metabolites (red – toxic, green – non-toxic).

Compound	T.E.S.T. Consensus[a]	Vega CAESAR[b]
Vor	0,57	1
VM1	0,63	0
VM2	0,46	0
VM3	0,53	1
VM4	0,56	1

a – mutagenic > 0,5; b – 0 = non-mutagenic, 1 = mutagenic

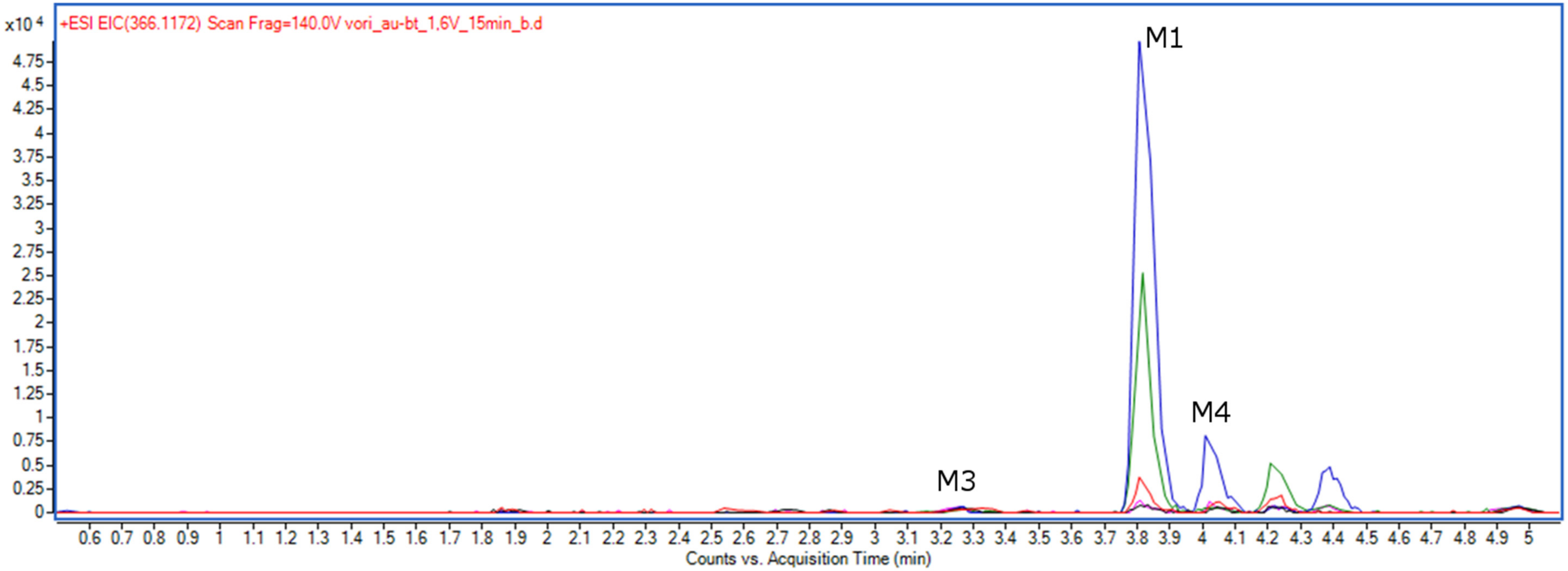

Figure S1. Overlaid EIC chromatograms of m/z 366.1172 obtained from the examined electrodes. The color-coding is as follows: blue corresponds to Fe(PH), pink to Cu(PH), green to Pt, red to Au, and black to GC SPEs. The optimal conditions for obtaining metabolites with a mass of m/z 366.1172 for the given electrodes were as follows: 1.0 V for the Fe(PH) electrode, 1.4 V for Cu(PH), 1.2 V for Pt, 1.6 V for Au, 1.4 V for GC. The chromatogram shows all the products resulting from monooxygenation, with. M1 appearing at a retention time of 3.80, M3 at 4.03, and M4 at 3.25. The peak at a retention time of 4.97 aligns with the retention time of the parent compound (in source clustering). Additionally, two products of electrochemical transformation are observable on the chromatogram at retention times of 4.23 and 4.40.

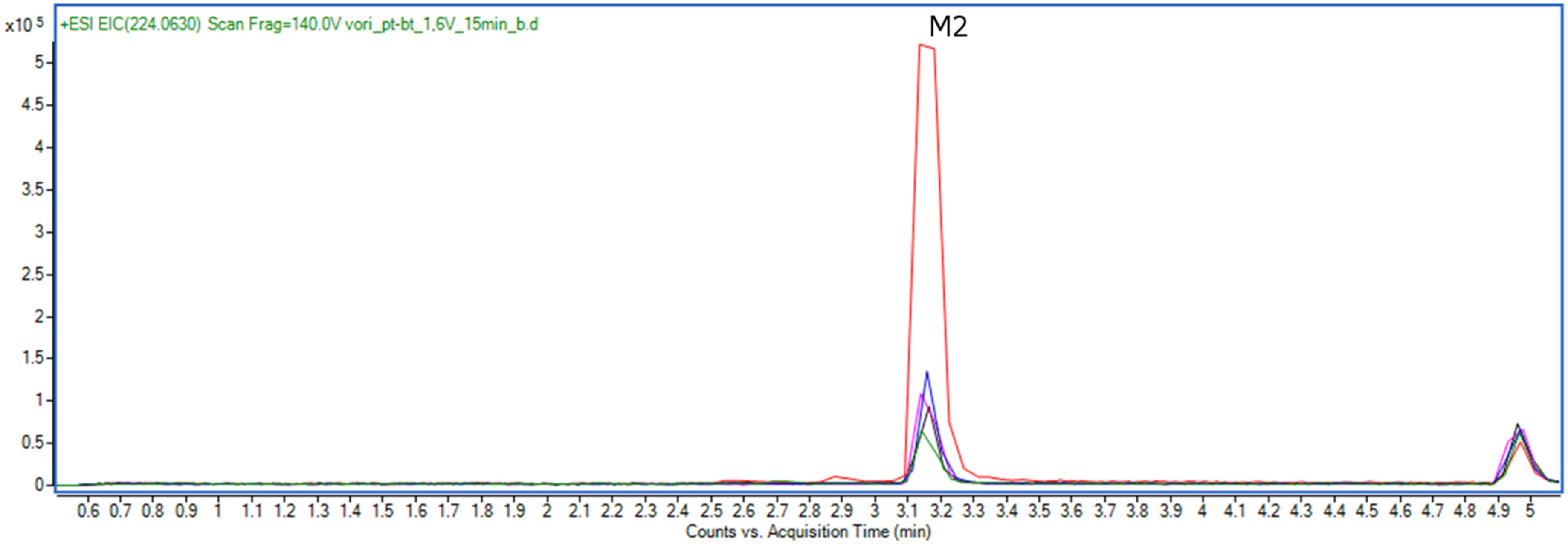

Figure S2. Overlaid EIC chromatograms of m/z 244.0630 obtained from the examined electrodes. The color-coding is as follows: blue corresponds to Fe(PH), pink to Cu(PH), green to Pt, red to Au, and black to GC SPEs. The optimal conditions for obtaining M2 for the given electrodes were as follows: 1.4 V for the Fe(PH) electrode, 1.4 V for Cu(PH), 1.6 V for Pt, 1.8 V for Au, 1.4V for GC. The peak belonging to M2 is visible in the chromatogram at a retention time of 3.15 min. The peak with a retention time of 4.97 aligns with the retention time of the parent compound undergoing decomposition in the ion source of mass spectrometry.

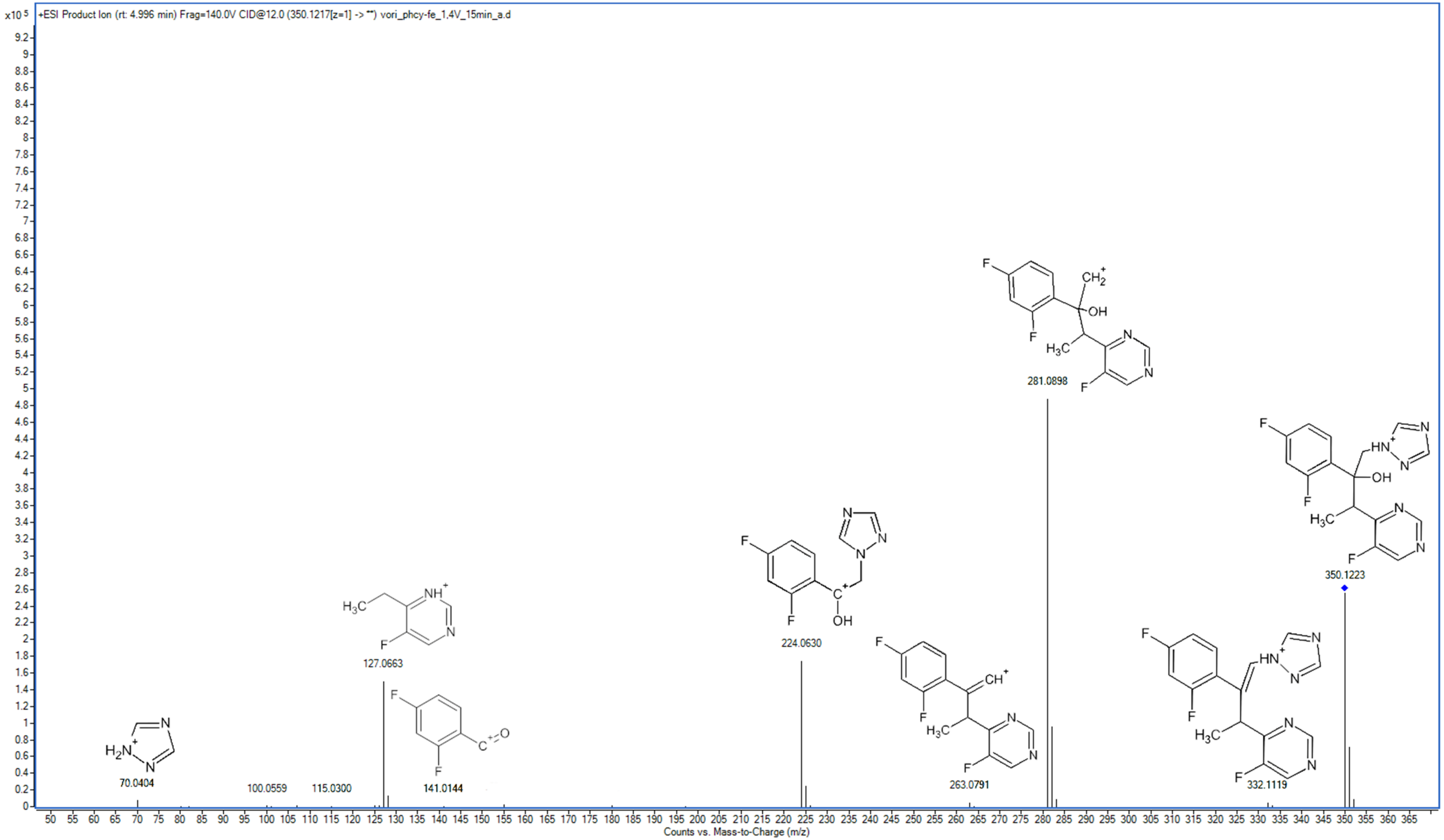

Figure S3. MS/MS spectrum and fragmentation pattern of voriconazole.

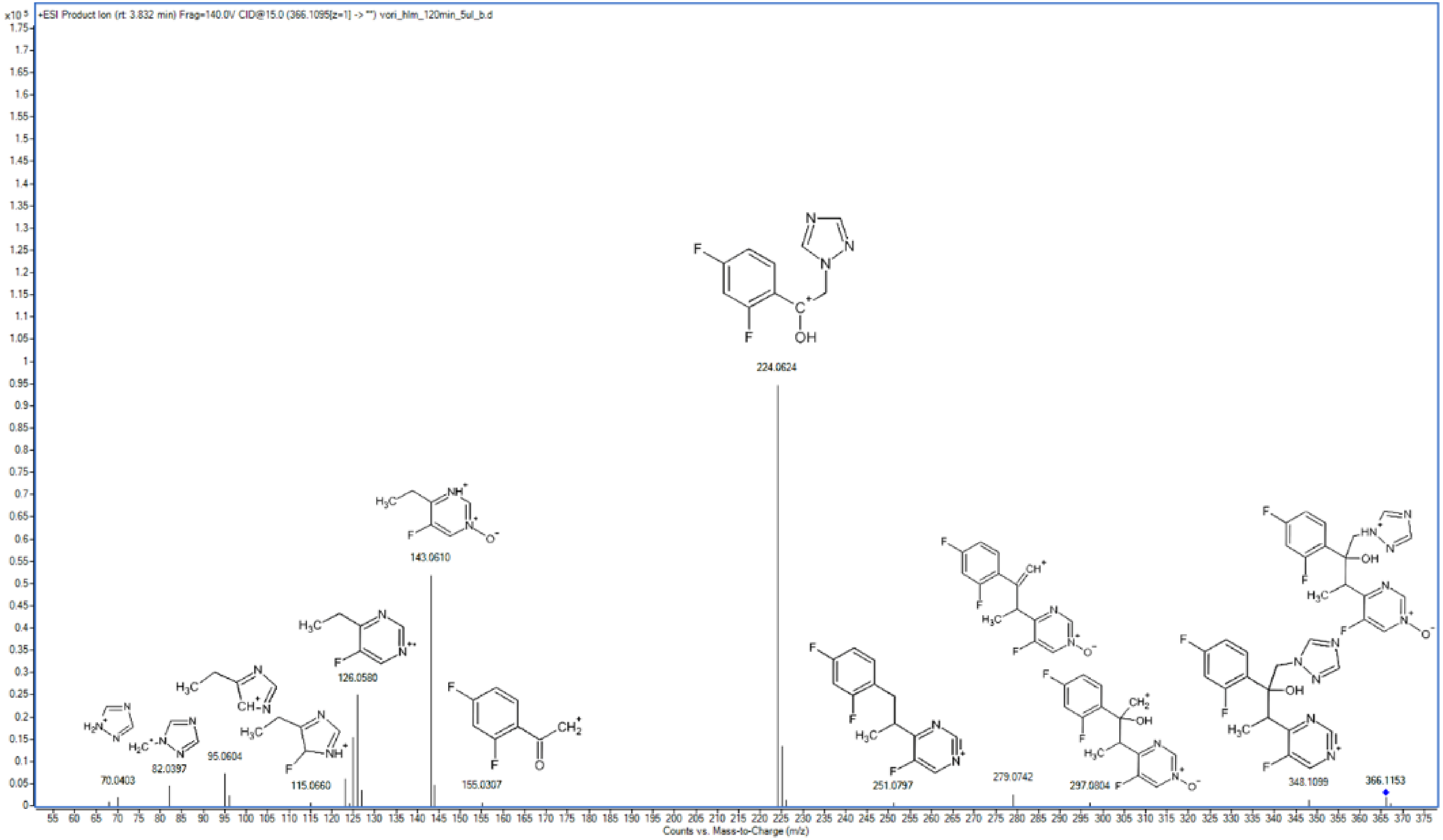

Figure S4. MS/MS spectrum and fragmentation pattern of M1.

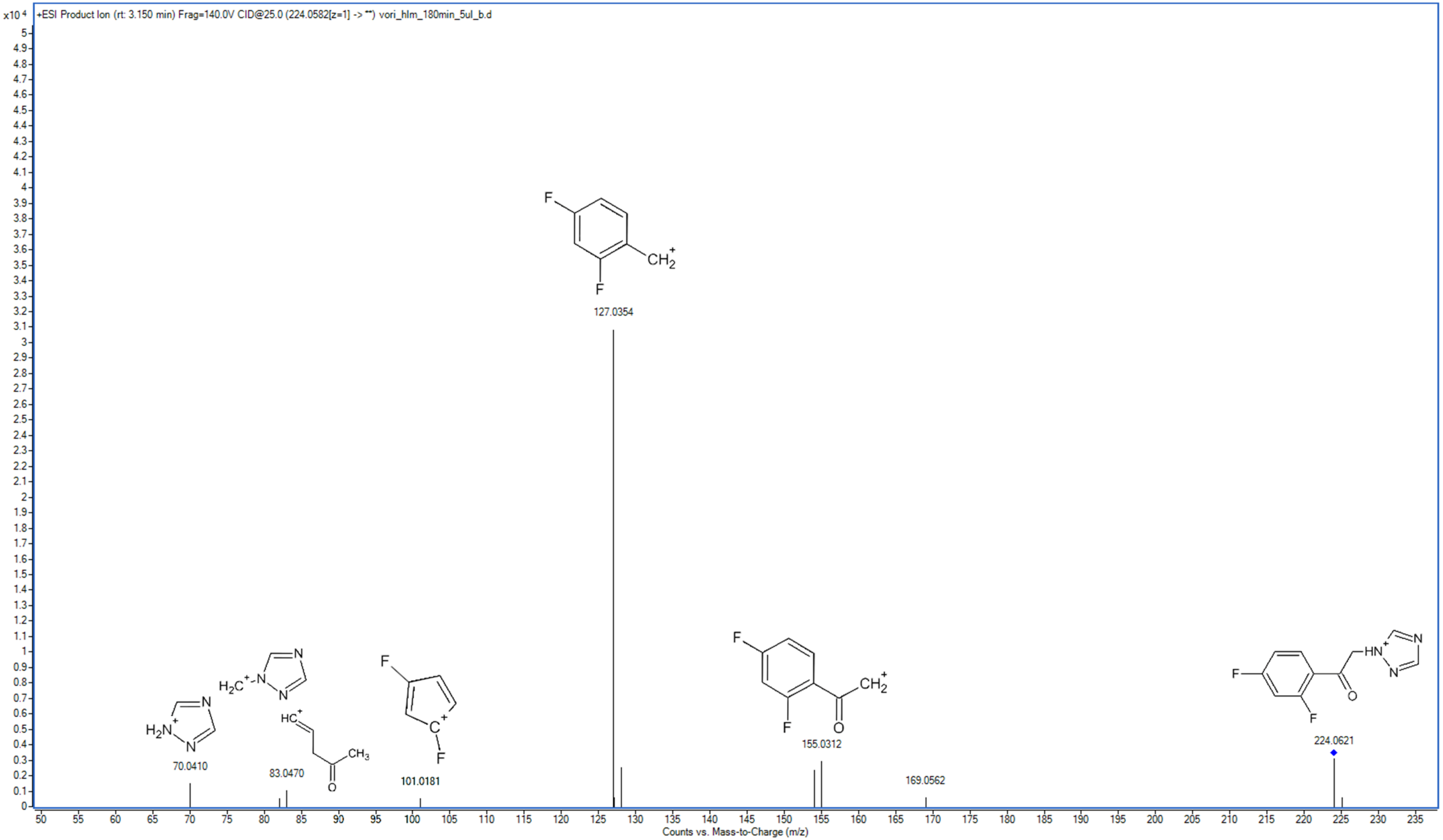

Figure S5. MS/MS spectrum and fragmentation pattern of M2.

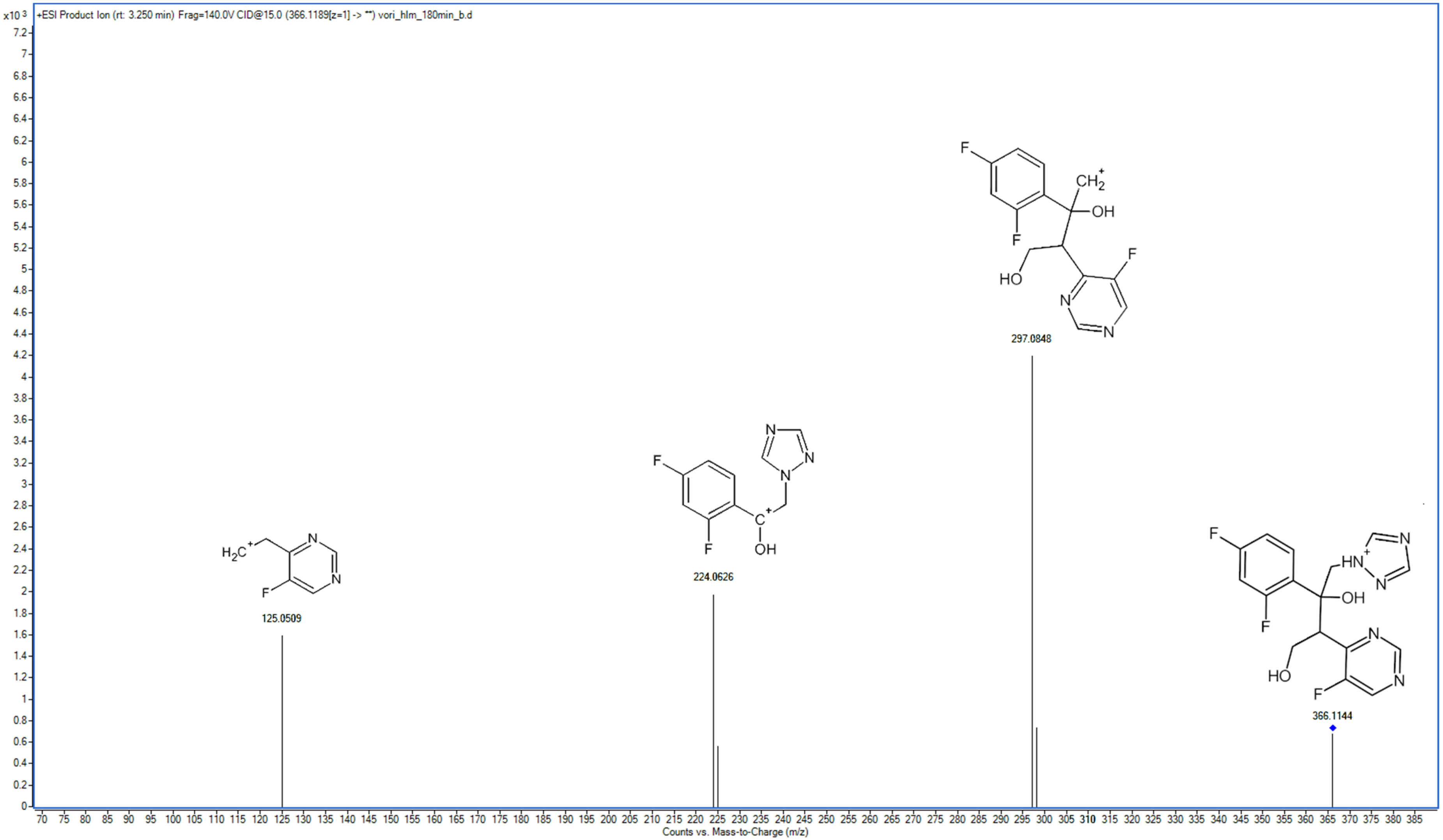

Figure S6. MS/MS spectrum and fragmentation pattern of M3.

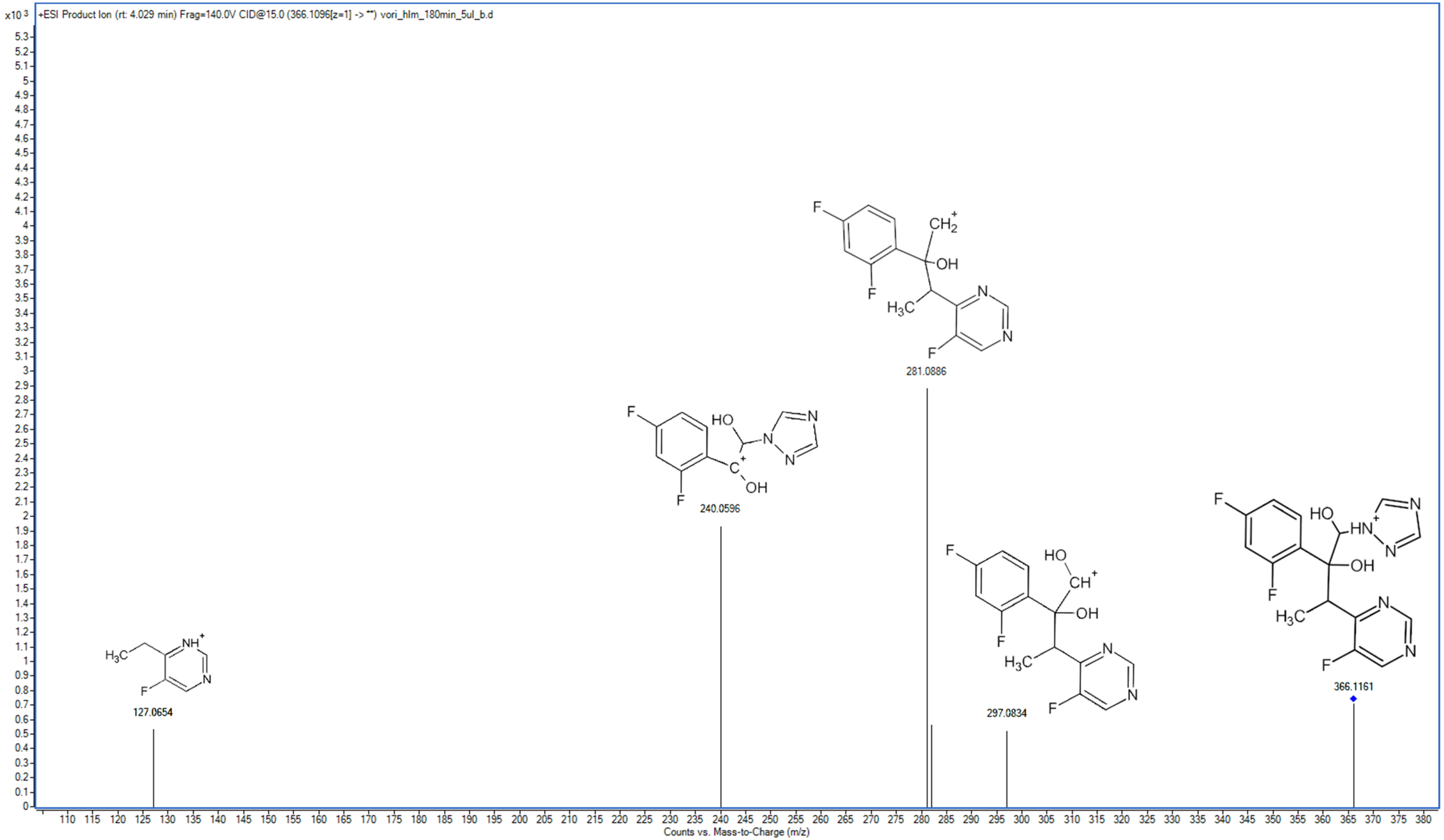

Figure S7. MS/MS spectrum and fragmentation pattern of M4.

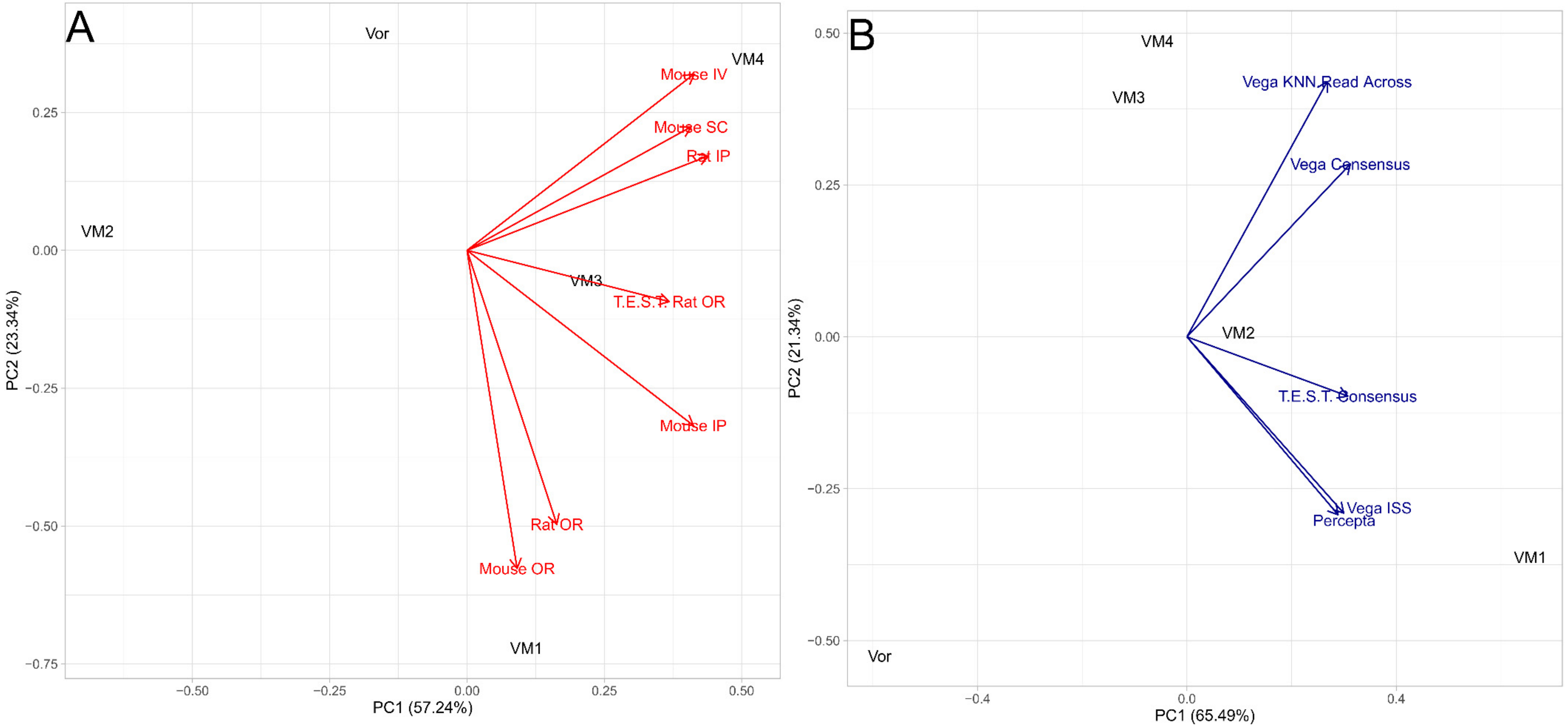

Figure S8. Comparison of toxicity of metabolites of voriconazole by PCA; IP – Intraperitoneal, IV – Intravenous, OR – Oral, SC – Subcutaneous.
